# Integrating Iris and Signature Traits for Personal Authentication Using User-Specific Weighting

**DOI:** 10.3390/s120404324

**Published:** 2012-03-29

**Authors:** Serestina Viriri, Jules R. Tapamo

**Affiliations:** 1 School of Computer Science, University of KwaZulu-Natal, Westville Campus, Durban 4000, South Africa; 2 School of Electrical, Electronic and Computer Engineering, Howard College, University of KwaZulu-Natal, Durban 4000, South Africa; E-Mail: tapamoj@ukzn.ac.za

**Keywords:** biometrics fusion, multi-modal biometrics, iris, signature, user-specific weighting

## Abstract

Biometric systems based on uni-modal traits are characterized by noisy sensor data, restricted degrees of freedom, non-universality and are susceptible to spoof attacks. Multi-modal biometric systems seek to alleviate some of these drawbacks by providing multiple evidences of the same identity. In this paper, a user-score-based weighting technique for integrating the iris and signature traits is presented. This user-specific weighting technique has proved to be an efficient and effective fusion scheme which increases the authentication accuracy rate of multi-modal biometric systems. The weights are used to indicate the importance of matching scores output by each biometrics trait. The experimental results show that our biometric system based on the integration of iris and signature traits achieve a false rejection rate (FRR) of 0.08% and a false acceptance rate (FAR) of 0.01%.

## 1. Introduction

Multi-modal biometric systems address the shortcomings of uni-modal systems. For instance, the problem of non-universality: it is possible for a subset of users to not possess a particular biometrics trait. For example, the feature extraction module of an iris authentication system may be unable to extract features from iris images associated with specific individuals, due to either the occlusion of the iris region of interest or poor quality of the images. Multi-modal systems ascertain that a *live* user is indeed authenticated. It is very difficult for intruders to circumvent multiple biometric traits simultaneously [1]. Thus, a challenge-response type of authentication can be facilitated using multi-biometric systems.

Furthermore, multi-modal biometric systems are expected to be more reliable due to the presence of multiple pieces of evidence [2]. Multi-modal systems should be able to meet the stringent performance requirements imposed by various applications [3]. In fact, research has proved that combining biometric techniques for human identification is more effective, but challenging [4]. Therefore, the problem of information fusion still needs attention in order to optimize the success rate of multi-modal biometric systems.

In this paper, a framework for modeling bi-modal biometric systems based on iris (a physiological trait) and the signature (a behavioral trait) for personal authentication is proposed. These two biometric traits are not correlated. Moreover, iris is proving to be one of the most reliable biometric traits while signatures continue to be widely used for personal authentication.

### 2. Related Work

Multi-modal biometrics was pioneered by Anil K. Jain; and there has been substantial research carried out in this area. A variety of biometric fusion schemes, which use classifiers, have been described in the literature to combine multiple biometric trait scores. These include majority voting, sum and product rules, k-NN classifiers, SVMs, and decision trees [4–6]. For instance, Ross *et al.* [1,7] combine the matching scores of the face, fingerprint and hand geometry using three different techniques, the sum rule, decision tree, and linear discriminant analysis. Experiments indicate that the fusion scheme using the sum rule with normalized scores gives the best performance. These results are further improved by learning user-specific matching thresholds and weights for individual biometric traits.

Other multi-modal biometric fusion approaches include: the HyperBF network approach used to combine the normalized scores of five different classifiers operating on the voice and face feature sets of an individual for identification [8]. Bigun *et al.* develop a statistical framework based on Bayesian statistics to integrate the speech (text-dependent) and face data of a user [9]. The estimated biases of each classifier is taken into account during the fusion process. Hong and Jain associate different confidence measures with the individual matchers when integrating the face and fingerprint traits of a user [3]. They also suggest an indexing mechanism wherein face information is used to retrieve a set of possible identities and the fingerprint information is then used to select a single identity. A commercial product called BioID [10] uses the voice, lip motion and face features of a user to verify identity. Brunelli and Falavigna also addressed an important aspect of fusion; the normalization of scores obtained from different domains [8]. Normalization maps the scores obtained from different ranges into a common range.

Although several score fusion techniques have been proposed in the literature, Ross *et al.* [11] grouped all of them into three main categories:

- **Density-based score fusion**: this technique estimates the conditional densities *p*(*s*∣*genuine*) and *p*(*s*∣*impositor*), where **s** = [*s*_1_, *s*_2_, …, *s_n_*] is the vector of matching scores, computes the probabilities *P*(*genuine*∣*s*) and *P*(*impositor*∣*s*), and can use the Bayesian rule to make a decision.
- **Transformation-based score fusion**: this approach transforms the match scores from different matchers into a common domain using normalization techniques.
- **Classifier-based score fusion**: learning pattern classifiers are used to determine the relationship between the vector of match scores, **s** = [*s*_1_, *s*_2_, …, *s_n_*] and the posteriori probabilities, *P*(*genuine*∣*s*) and *P*(*impositor*∣*s*).

In this paper, an enhanced user-specific weighting technique is proposed, which is based on the different degrees of importance for different traits of an individual to integrate the physiological trait, the *iris* and behavioral trait, the *signature*. The user-specific weights for individual biometric traits are calculated based on the score of each biometric trait of an individual user. The proposed approach is an alternative to the estimation of user-specific weights by exhaustive search.

The rest of the paper is structured as follows: Section 3 explores various fusion techniques for combining biometric traits; Section 4 describes an overall multi-modal biometrics system; Section 5 describes the weighting techniques and normalization strategies; Section 6 presents experimental results; and Section 7 draws the conclusions and future work.

## 3. Multi-Modal Biometrics System

Multi-modal biometric systems are based on the consolidation of information presented by multiple evidences that stem from multiple traits. Some of the limitations imposed by uni-modal biometric systems (that is, biometric systems that rely on the evidence of a single biometric trait) can be overcome by using multiple biometric modalities [4,8,9]. Such systems, known as multi-biometric systems, are expected to be more reliable due to the presence of multiple, fairly independent pieces of evidence.

A variety of factors should be considered when designing a multi-biometric system. These include the choice and number of biometric traits; the level in the biometric system at which information provided by multiple traits should be integrated; the methodology adopted to integrate the information; and the cost *vs.* matching performance trade-off.

**Figure 1.**
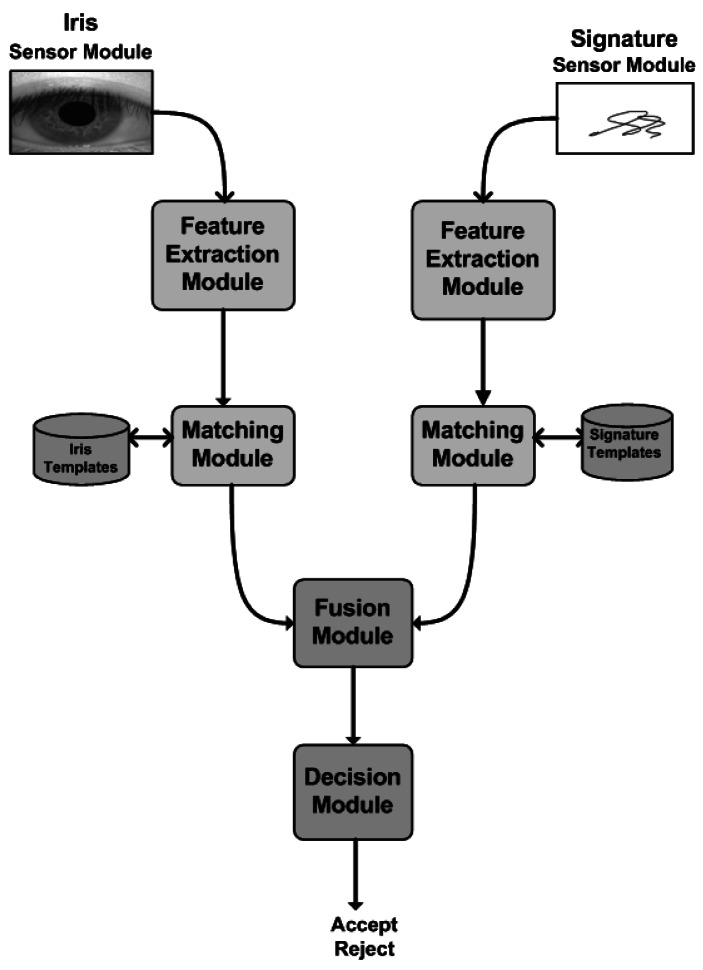
Multi-modal Biometrics System (Iris & Signature).

A simple multi-modal biometrics system has five important components as depicted in Figure 1, in which different biometric traits are fused at match score level:

1. **Sensor module**, acquires the biometric data of an individual. An example is the ePadInk tablet that captures the signature.
2. **Feature extraction module**, the acquired biometric data is processed to extract distinctive feature values.
3. **Matching module**, the extracted feature values are compared against those in the template by generating a matching score.
4. **Fusion module**, combines the biometric trait scores.
5. **Decision module**, a claimed identity is either accepted or rejected based on the fusion matching score generated in the fusion module.

## 4. Fusion in Biometrics

There are various levels of fusion for combining biometric traits. The three possible levels of fusion are [1, 11]:

1. **Fusion at the sensor level**: The consolidation of evidence captured by multiple sources of the input data before feature extraction.
2. **Fusion at the feature extraction level**: The data obtained from each sensor is used to compute a feature vector. If the features extracted from one biometric trait are independent of those extracted from the other, it is better to concatenate the two vectors into a single new vector. The new feature vector now has a higher dimensionality and represents a person's identity in a different hyperspace. Feature reduction techniques may be employed to extract useful features from the larger set of features.
3. **Fusion at the matching score level**: Each subsystem provides a matching score indicating the proximity of the feature vector with the template vector. These scores can be combined to assert the veracity of the claimed identity. Fusion techniques such as logistic regression may be used to combine the scores reported by different sensors. These techniques attempt to minimize the FRR for a given FAR [12].
4. **Fusion at the rank level**: The consolidation of the ranks output by individual biometric subsystems in order to drive a consensus rank for each identity [11].
5. **Fusion at the decision level**: Each sensor can capture multiple biometric data and the resulting feature vectors are individually classified into the two classes: *accept* or *reject*. A majority vote scheme, such as that employed in [13] can be used to make the final decision.

## 5. Integrating Iris and Signature Traits

A brief description of the two biometric traits used in this research work is given below.

### 5.1. Iris Recognition

Iris recognition is proving to be one of the most reliable biometric traits for personal identification since iris patterns have stable, invariant and distinctive features. Several techniques have been proposed for iris segmentation, coding and matching. The most common approach used in iris recognition is to generate feature vectors corresponding to individual iris images and perform iris matching based on some distance measures [14,15]. In this research work, an algorithm that detects the largest non-occluded rectangular part of the iris as region of interest (ROI) is used [16]. A cumulative-sum-based grey change analysis technique is applied to the ROI to extract features for recognition [17]. Then, the Hamming Distance is computed as the iris matching score.

### 5.2. Signature Verification

Signature continues to be an important biometric trait because it remains widely used primarily for authenticating the identity of human beings. An efficient text-based directional signature recognition algorithm which verifies signatures, even when they are composed of symbols and special unconstrained cursive characters which are superimposed and embellished is used [18]. This algorithm extends the character-based signature verification technique. The text-based directional algorithm integrates the direction information extracted from the structure of the whole signature text image contours with the transition information between background and foreground pixels in the signature text image. The extracted features represent the distinguishing cursive handwriting styles. Then, the Mahalanobis Distance is computed as the signature matching score.

### 5.3. Combining Iris and Signature Traits

The iris and signature traits are fused at the matching score level, where the matching scores output of each of these two traits are weighted and combined. Fusion at the matching score level is usually preferred, as it is relatively easy to access and combine the scores presented by the different modalities [4]. There are two distinct approaches for the match score level fusion: the *classification problem* approach [6], where a feature vector is constructed using the matching scores output by the individual matchers, and the *combination problem* approach, where the individual matching scores are combined to generate a single scalar score, which is then used to make the final decision. The literature shows that the *combination* approach performs better than the *classification* approach [1]; hence, it is adopted in this paper. The combining process is summarized in Algorithm 1.

**Algorithm 1** Fusion of Iris and Signature Traits.1:**for** each fusion per User **do**2: **for** each User **do**3:   **if** iris **then**4:     *S_iris_* ← *HammingDistance* {//Iris Score Generation}5:   **else**6:     *S_sig_* ← *MahalanobisDistance* {//Signature Score Generation}7:   **end if**8: **end for**9: **for** each score **do**10:   **if**
*S_iris_*
**then**11:     
$S_{\text{iris}}^{\prime }\leftarrow \text{Normalization}(S_{\text{iris}})$12:   **else**13:     
$S_{\text{sig}}^{\prime }\leftarrow \text{Normalization}(S_{\text{sig}})$14:   **end if**15: **end for**16: **for** each normalized score **do**17:   **if**
$S_{\text{iris}}^{\prime }$
**then**18:     
$W_{\text{iris}}\leftarrow \text{Weighting}(S_{\text{iris}}^{\prime })$19:   **else**20:     
$W_{\text{sig}}\leftarrow \text{Weighting}(S_{\text{sig}}^{\prime })$21:   **end if**22: **end for**23: 
$S_{\text{fus}}\leftarrow W_{\text{iris}}S_{\text{iris}}^{\prime }+W_{\text{sig}}S_{\text{sig}}^{\prime }$24:**end for**

#### Score Generation

Iris matching scores are computed from string iris feature codes extracted by the cumulative-sum-based grey change analysis technique. To verify the similarity of two iris codes, Hamming Distance (HD) based on the matching algorithm [19] is used. The smaller the HD, the higher the similarity of the compared iris codes. The HD denotes the iris raw matching score, *S_iris_*, which is computed as:

(1) $$S_{\text{iris}}=\frac{1}{2N}[(\sum_{i=1}^{N}A_{h}(i)⊕B_{h}(i))+(\sum_{i=1}^{N}A_{v}(i)⊕B_{v}(i))]\;\text{only when}\;A_{h}(i)\neq 0\wedge B_{h}(i)\neq 0,A_{v}(i)\neq 0\wedge B_{v}(i)\neq 0$$

where *A_h_*(*i*) and *A_v_* (*i*) denote the enrolled iris code over horizontal and vertical directions, respectively, *B_h_*(*i*) and *B_v_*(*i*) denote the new input iris code over the horizontal and vertical directions respectively. *N* is the total number of cells, and ⊕ is the XOR operator.

Signature matching scores are generated from the signature feature vectors. To verify the similarity of two signatures, Mahalanobis Distance (MD) based on correlations between signatures is used. It differs from Euclidean distance in that it takes into account the correlations of the data set and is scale-invariant. The smaller the MD, the higher the similarity of the compared signatures. The MD denotes the signature raw matching score, *S_sig_*, which is computed as in Equation (2).

(2) $$S_{\text{sig}}(\vec{x},\vec{y})=\sqrt{{\left(\vec{x}-\vec{y}\right)}^{T}S^{-1}(\vec{x}-\vec{y})}$$

where *x⃗* and *y⃗* denote the enrolled feature vector and the new signature feature vector to be verified, with the covariance matrix *S*.

#### Score Normalization

Given a set of *n* raw matching scores {*S_k_*}, *k* = 1,2, …, *n*, the corresponding normalized scores $S_{k}^{\prime }$ are given by:

- *Min-max normalization*: retains the original distribution of scores and maps all the scores into the [0, 1] range.
  (3) $$S_{k}^{\prime }=\frac{S_{k}-\min(\{S_{k}\})}{\min(\{S_{k}\})-\min(\{S_{k}\})}$$
  where *min*({*S_k_*}) and max({*S_k_*}) are the minimum and maximum, respectively, of the given set {*S_k_*} of matching scores.
- *Z-score normalization*: transforms the scores to a distribution with arithmetic mean of 0 and standard deviation of 1.
  (4) $$S_{k}^{\prime }=\frac{S_{k}-\mu }{\sigma }$$
  where *μ* and *σ* are the mean and standard deviation, respectively, of the set {*S_k_*}.
- *Tanh normalization*: is a robust statistical technique [20] which maps the raw scores into the [0, 1] range.
  (5) $$S_{k}^{\prime }=\frac{1}{2}\left\{\tanh\left(0.01\left(\frac{S_{k}-\mu }{\sigma }\right)\right)+1\right\}$$
  where *μ* and *σ* are the mean and standard deviation, respectively, of {*S_k_*}.

**Figure 2.**
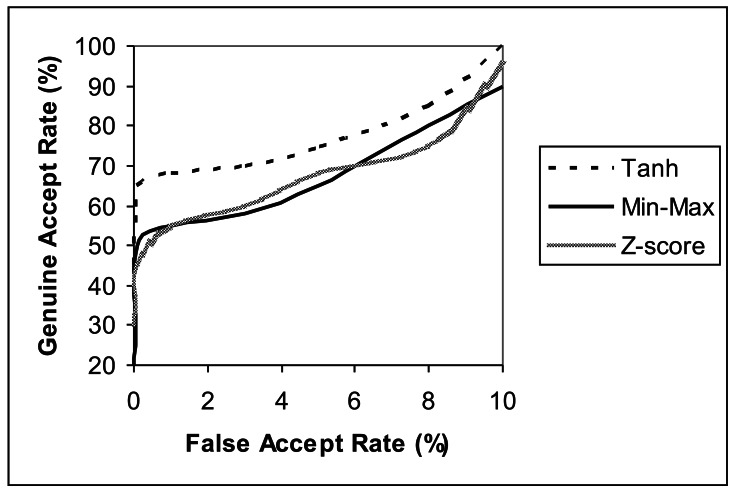
ROC curves showing the performance of each of the three normalization techniques on the Iris trait.

The ROC curves depicting the performance of the individual score normalization techniques implemented on iris biometrics trait is shown in Figure 2. The *CASIA* iris database [21] is used for comparing and contrasting these normalization algorithms. A similar experiment was conducted on the signature trait using the *GPDS* signature database [22], and it obtained comparable results to the iris trait. As a result, *tanh normalization* technique performs better than *min-max* and *Z-score* techniques.

#### Score Weighting

Let $s_{\text{iris}}^{\prime }$ and $s_{\text{sig}}^{\prime }$ be the normalized scores of the iris and signature traits, respectively. The fusion score, *s_fus_* is computed as

(6) $$s_{\text{fus}}=w_{\text{iris}}s_{\text{iris}}^{\prime }+w_{\text{sig}}s_{\text{sig}}^{\prime }$$

where *w_iris_* and *w_sig_* are the *weights* associated with the degrees of importance of the two traits per individual, and

(7) $$w_{\text{iris}}+w_{\text{sig}}=1$$

Different iris scores and signature scores are given different degrees of importance for different users. For instance, by reducing the weight *w_iris_* of an occluded iris and increasing the weight *w_sig_* associated with the signature trait, the false reject error rate of the particular user can be reduced. The biometric system learns user-specific parameters by observing system performance over a period of time [4]. Two techniques are used to compute the user-specific weights: *an exhaustive search technique*, and *a user-score-based technique*.

#### The Exhaustive Search Technique

Let $w_{\text{iris}}^{i}$ and $w_{\text{sig}}^{i}$, be the weights associated with the *i^th^* user in the database. The algorithm operates on the training set as follows [7]:

- For the *i^th^* user in the database, vary weights $w_{\text{iris}}^{i}$ and $w_{\text{sig}}^{i}$ over the range [0, 1], with the constraint $w_{\text{iris}}^{i}+w_{\text{sig}}^{i}=1$. Compute $s_{\text{fus}}^{i}=w_{\text{iris}}^{i}s_{\text{iris}}^{\prime i}+w_{\text{sig}}^{i}s_{\text{sig}}^{\prime i}$. This computation is performed over all scores associated with the *i^th^* user.
- Choose that set of weights that minimizes the total error rate. The total error rate is the sum of the false acceptance and false rejection rates pertaining to this user.

The set of weights, $\left\{w_{\text{iris}}^{i},w_{\text{sig}}^{i}\right\}$, that minimizes the total error rate, with the constraint $w_{\text{iris}}^{i}+w_{\text{sig}}^{i}=1$, does not necessarily associate the degrees of importance for iris and signature biometric traits of the *i^th^* individual in the fusion score: $s_{\text{fus}}^{i}=w_{\text{iris}}^{i}s_{\text{iris}}^{\prime i}+w_{\text{sig}}^{i}s_{\text{sig}}^{\prime i}$. An alternative user-score-based weighting technique, which computes the weights, $\left\{w_{\text{iris}}^{i},w_{\text{sig}}^{i}\right\}$, by associating them with the degrees of importance for iris and signature biometric traits, respectively, is proposed. In this method, the weights, $\left\{w_{\text{iris}}^{i},w_{\text{sig}}^{i}\right\}$, which are not constrained to $w_{\text{iris}}^{i}+w_{\text{sig}}^{i}=1$, are computed in consideration of how close the scores $s_{\text{iris}}^{\prime i}$ and $s_{\text{sig}}^{\prime i}$ are to the thresholds of the iris and signature traits, respectively. The user-score-based weighting technique is described below.

#### The User-Score-Based Technique

Let $s_{\text{iris}}^{\prime i}$ and $s_{\text{sig}}^{\prime i}$ be the normalized scores associated with the *i^th^* user in the database, and *τ*_1_ and *τ***_2_** are the thresholds of the iris and signature traits, respectively. The preliminary weights $w_{\text{iris}}^{\prime i}$ and $w_{\text{sig}}^{\prime i}$ per trait are computed as

(8) $$w_{\text{iris}}^{\prime i}=\frac{s_{\text{iris}}^{\prime i}}{\tau_{1}+s_{\text{iris}}^{\prime i}}$$

and

(9) $$w_{\text{sig}}^{\prime i}=\frac{s_{\text{sig}}^{\prime i}}{\tau_{2}+s_{\text{sig}}^{\prime i}}$$

where $w_{\text{iris}}^{\prime i}$ and $w_{\text{sig}}^{\prime i}$ are the initial weights associated with the iris and signature, respectively, **without** the constraint $w_{\text{iris}}^{\prime i}+w_{\text{sig}}^{\prime i}=1$. These weights are assigned to the scores, $s_{\text{iris}}^{\prime i}$ and $s_{\text{sig}}^{\prime i}$ after analyzing how close or farther away the scores are from their respective thresholds, *τ*_1_ and *τ*_2_. Then, the fusion weights for the *i^th^* user are computed respectively, for the iris and signature as

(10) $$w_{\text{iris}}^{i}=\frac{w_{\text{iris}}^{\prime i}}{w_{\text{iris}}^{\prime i}+w_{\text{sig}}^{\prime i}}$$

(11) $$w_{\text{sig}}^{i}=\frac{w_{\text{sig}}^{\prime i}}{w_{\text{iris}}^{\prime i}+w_{\text{sig}}^{\prime i}}$$

**with** the constraint $w_{\text{iris}}^{i}+w_{\text{sig}}^{i}=1$, and the fusion score is computed in Equation (12).

(12) $$s_{\text{fus}}^{i}=w_{\text{iris}}^{i}s_{\text{iris}}^{\prime i}+w_{\text{iris}}^{i}s_{\text{sig}}^{\prime i}$$

#### Score Fusion

The dual *ν*-Support Vector Machine (2*ν*-SVM) fusion algorithm [23] is used to integrate the matching scores of the iris *s_iris_* and signature *s_sig_*, together with their corresponding weights, *w_iris_* and *w_sig_*. The weighted iris matching score *m_iris_* is defined as

(13) $$m_{\text{iris}}=s_{\text{iris}}\times w_{\text{iris}}$$

and the weighted signature score *m_sig_* is defined as

(14) $$m_{\text{sig}}=s_{\text{sig}}\times w_{\text{sig}}$$

The weighted matching scores and their labels are used to train the 2*ν*-SVM for bimodal fusion. Let the training data be

(15) $$Z_{\text{iris}}=(m_{\text{iris}},y)$$

and

(16) $$Z_{\text{sig}}=(m_{\text{sig}},y)$$

where *y* ∈ {+1, −1}, such that +1 represents the genuine class and −1 represents the impostor class. The 2*ν*-SVM error parameters are calculated using Equation (17) and (18).

(17) $$\nu_{+}=\frac{n_{+}}{n_{+}+n_{-}}$$

(18) $$\nu_{-}=\frac{n_{-}}{n_{+}+n_{-}}$$

where *n_+_* and *n*_−_ are the number of genuine and impostor, respectively. The training data is mapped into a higher dimension feature space such that *Z* → *φ* (*Z*), where *φ*(.) is the mapping function. The optimal hyperplane separates the data into two different classes in the higher dimensional feature space.

In the classification phase, the bi-modal fusion matching score *s_fus_* is computed in Equation (19),

(19) $$s_{\text{fus}}=f_{\text{iris}}(m_{\text{iris}})+f_{\text{sig}}(m_{\text{sig}})$$

where

(20) $$f_{\text{iris}}(m_{\text{iris}})=a_{\text{iris}}\varphi (m_{\text{iris}})+b_{\text{iris}}$$

(21) $$f_{\text{sig}}(m_{\text{sig}})=a_{\text{sig}}\varphi (m_{\text{sig}})+b_{\text{sig}}$$

where *a_iris_, a_sig_, b_iris_* and *b_sig_* are parameters of the hyperplane. The solution of Equation (19) is the signed distance of *s_fus_* from the separating hyperplane given by the two 2*ν*-SVM for the two biometric modalities. The decision function defined in Equation (22) verifies the identity.

(22) $$\text{Decision}(s_{\text{fus}})=\{\begin{array}{cc} \text{Accept}, & \text{if}\;s_{\text{fus}}>0\\ \text{Reject}, & \text{otherwise} \end{array}$$

## 6. Experimental Results and Discussions

The performance of the investigated bi-modal biometrics system is evaluated by calculating its false acceptance rate (FAR) and false rejection rate (FRR) at various thresholds. These two factors are integrated together in a receiver operating characteristic (ROC) curve that plots the FRR or the genuine acceptance rate (GAR) against the FAR at different thresholds. The FAR and FRR are computed by generating all possible genuine and impostor matching scores and then setting a threshold for deciding whether to accept or reject a match.

The bi-modal database used in the experiments was constructed by merging *CASIA* iris database [21] with *GPDS* signature database [22]. An alternative bi-modal database was constructed from *CASIA* iris database and a database created from signatures captured using the ePadInk tablet. Seven iris images of the same user were obtained from a set of 50 users from the *CASIA* database. Fifteen signatures (ten genuine and 5 forgeries) were obtained from a different set of 50 users from the *GPDS* database, and another set of signatures were captured using ePadInk tablet. The mutual independence assumption of the iris and signature biometric traits allows us to randomly pair the users from the two different data sets. In this way, two bi-modal databases consisting of 50 users were constructed, either from *CASIA* with *GPDS*, or *CASIA* with signatures captured using ePadInk tablet.

Firstly, the matching scores of the iris and signature traits are computed as defined in Equations (1) and (2). These matching scores are normalized and weighted as defined in subsections of 5.3. Various normalization techniques were investigated. The ROC curves depicting the performance of the individual score normalization techniques is shown in Figure 2. The *Tanh Normalization* technique performs better than the *Min-Max* and *Z-Score* techniques.

Table 1 shows the scores for the iris and signature biometric traits, and their respective weights, for the sample of ten different individuals. The raw scores are normalized by the tanh technique, and the weights are computed using Equations (10) and (11). For instance, from Table 1, we observe that for user 5, $W_{1}^{i}=0.83$, a high weight attached to the iris trait, possibly due to the blurred iris. This demonstrates the importance of assigning user-specific weights to the individual biometric trait.

**Figure 3.**
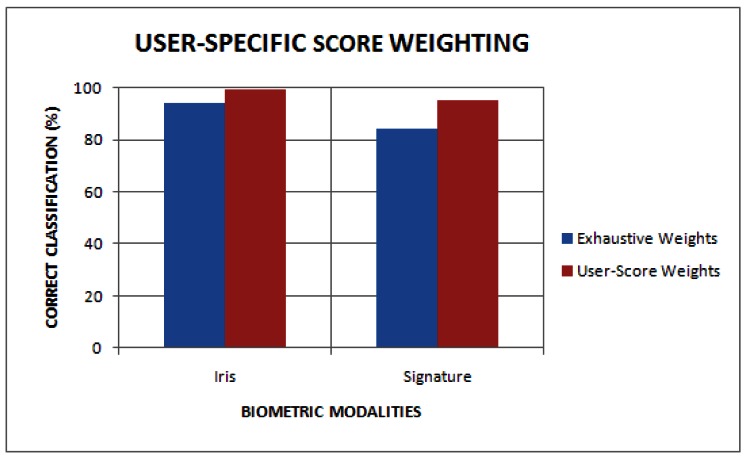
Average true positive rate of the iris and signature Modalities.

Figure 3 shows the average true positive rates achieved by the exhaustive search technique and the user-score-based approach, respectively, on uni-modal biometric traits based on iris and signature. The exhaustive search technique obtained true positive rates of 92.4% and 82.0% on the iris and signature traits, respectively. The user-score-based approach obtained true positive rates of 99.25% and 94.0% on the iris and signature traits, respectively. The overall average true positive rate achieved by the user-score-based is 99.6%. Therefore, the results show an improvement in accuracy when the user-score-based weighting technique is used.

### 6.1. Validation of the User-Score-Based Weighting Algorithm

**Figure 4.**
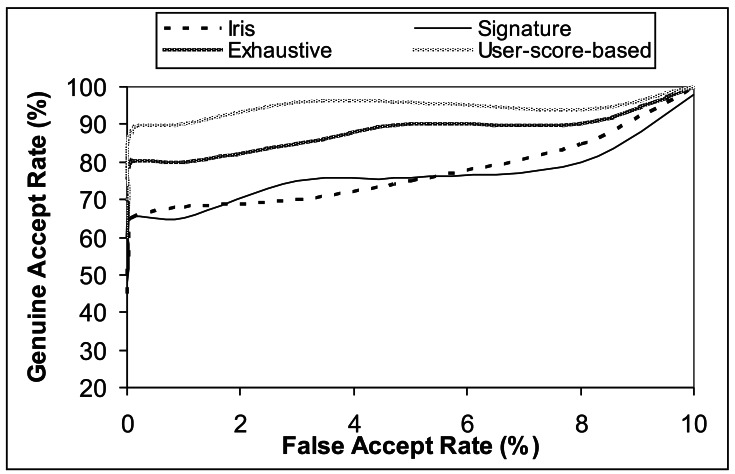
Tanh normalized-based ROC curves showing the performance of using Iris, Signature, Iris + Signature (Exhaustive), and Iris + Signature (User-score-based).

The ROC curves in Figure 4, show the performance of the uni-modal biometric traits based on iris and signature, respectively, and the 2*ν*-SVM fused based bi-modal traits weighted by the exhaustive search technique and the user-score-based approach, respectively. The overall results show an improvement in performance when scores are combined using the user-score-based weighting technique. For a given FAR of 0.01%, user-score-based weighting achieve a very low FRR of 0.08%, compared to exhaustive search weighting with a FRR of 0.75%, as shown in Table 2.

The user-score-based weighting algorithm computes the weights of the iris and signature traits by analyzing how close the two matching scores are to their respective thresholds, hence associating the weights with the different degrees of importance for the bi-modal biometric traits involved. Comparatively, the exhaustive search weighting technique calculates weights that simply minimize the total error rate. This minimum error rate (the sum of FAR and FRR) does not necessarily reflect the different degrees of importance for the bi-modal biometric traits fused.

### 6.2. Comparison with Existing Bi-Modal Biometric Systems

Table 3 shows the performance of the user-score-based weighted 2*ν*-SVM fusion algorithm, compared to other bi-modal biometric fusion algorithms in the literature. The quality based sum-rule [23] obtained an accuracy rate of 97.39%, when used to fuse the face and iris modalities, whereas the fusion of the iris and signature modalities based on the user-score-based weighted 2*ν*-SVM technique achieves an accuracy rate of 99.6%.

## 7. Conclusions

In this paper, an enhanced user-specific weighting technique of integrating a physiological biometrics trait, the *iris*, with a behavioral trait, the *signature*, is proposed. The proposed user-score-based approach calculates weights for each biometrics trait per user in proportion to the scores of the biometric traits of the same user. This enhanced user-specific weighting improves the accuracy rate of bi-modal biometric systems by reducing false reject rate (FRR) on a low false accept rate (FAR). Experimental results show that the proposed approach achieved a minimal FRR of 0.08% on a FAR of 0.01%. Further investigation of the effect of the proposed approach with other different biometric modalities is envisaged.

## Figures and Tables

**Table 1.** User-specific Scores and Weights of different traits for 10 users.

**User**	**Iris Score**	**Signature Score**	**Normalized Iris Score**	**Normalized Signature Score**	**Iris Weight**	**Signature Weight**
1	0.192	0.001	0.487	0.488	0.80	0.20
2	0.277	0.001	0.490	0.488	0.86	0.14
3	0.625	2.054	0.505	0.505	0.50	0.50
4	0.446	2.438	0.506	0.496	0.44	0.56
5	0.232	0.005	0.486	0.492	0.83	0.17
6	0.473	2.383	0.498	0.507	0.47	0.53
7	0.071	0.028	0.484	0.493	0.67	0.33
8	0.522	2.474	0.505	0.507	0.47	0.53
9	0.366	1.358	0.497	0.502	0.48	0.52
10	0.451	1.774	0.502	0.506	0.50	0.50

**Table 2.** Exhaustive search *vs.* User-score-based technique.

**Weighting Technique**	**FAR (%)**	**FRR (%)**
Exhaustive search	0.01	0.75
User-score-based	0.01	0.08

**Table 3.** Comparative table of the weighted based fusion algorithms.

**Biometric Modalities**	**Weighted Fusion Algorithm**	**Verification Accuracy (%)**
Face + Iris	Quality based Sum-rule [23]	97.39
Face + Speech	k-NN based fusion [24]	99.72
Face + Iris	Quality based [23]	98.91
Iris + Signature	User-Score-based Weighted 2*ν*-SVM	99.6
